# Monitoring Frameworks for Universal Health Coverage: What About High-Income Countries?

**DOI:** 10.15171/ijhpm.2019.21

**Published:** 2019-04-22

**Authors:** Nicole Bergen, Arne Ruckert, Ronald Labonté

**Affiliations:** ^1^ Faculty of Health Sciences, University of Ottawa, Ottawa, ON, Canada.; ^2^ Faculty of Medicine, School of Epidemiology and Public Health, University of Ottawa, Ottawa, ON, Canada.; ^3^ Canada Research Chair, Globalization and Health Equity, Faculty of Medicine, School of Epidemiology and Public Health, University of Ottawa, Ottawa, ON, Canada.

**Keywords:** Universal Health Coverage, High-Income Countries, Sustainable Development Goals, Monitoring

## Abstract

Implementing universal health coverage (UHC) is widely perceived to be central to achieving the Sustainable Development Goals (SDGs), and is a work program priority of the World Health Organization (WHO). Much has already been written about how low- and middle-income countries (LMICs) can monitor progress towards UHC, with various UHC monitoring frameworks available in the literature. However, we suggest that these frameworks are largely irrelevant in high-income contexts and that the international community still needs to develop UHC monitoring framework meaningful for high-income countries (HICs). As a first step, this short communication presents preliminary findings from a literature review and document analysis on how various countries monitor their own progress towards achieving UHC. It furthermore offers considerations to guide meaningful UHC monitoring and reflects on pertinent challenges and tensions to inform future research on UHC implementation in HIC settings.

## Background

Achieving universal health coverage (UHC) by 2030 is embedded in the Sustainable Development Goals (SDGs), and is a work program priority for the World Health Organization (WHO) over the next decade. UHC is considered by the WHO in many of its public statements to be foundational to accomplishing most of the other health related SDGs.^1^ But what does achieving UHC mean for high-income countries (HICs), with a recent report suggesting that nearly all Organisation for Economic Co-operation and Development countries have already done so and can tick this target off of their to-do lists?^2^ (A claim that we address and dispute throughout this article). Moreover, given the emphasis the SDGs place on measuring country progress, one of the present lacunae is whether the currently agreed-upon UHC indicators are relevant to improving equitable health system access in HICs. In this short communication, we argue that UHC achievement remains an important goal for all countries, including HICs, but one that also requires consideration to indicators appropriate to the contexts of wealthier countries with already well-developed health systems.

The WHO defines UHC as all individuals and communities receiving the health services they need without suffering financial hardship, with coverage for the full spectrum of essential, quality health services, from health promotion to prevention, treatment, rehabilitation, and palliative care.^3^ UHC incorporates 3 dimensions of: health service coverage (what services are covered), financial protection (how much do people have to pay to receive services), and population coverage (who is covered). Although much attention on UHC is focussed on its progressive realization in low- and middle-income countries (LMICs), the potential health equity implications of UHC are equally resonant in HICs. Like LMICs, for HICs, the pursuit of UHC implies on-going improvement of health service provision and equitable access with no end point. This raises questions about how such improvements in HICs should be monitored, especially given that many HICs in response to the 2008 global financial crisis implemented austerity measures that could weaken, rather than strengthen, UHC. Amongst these reforms were efforts to: encourage those who could afford to do so to opt out of the public health care system by purchasing private insurance; limit publicly funded benefit packages and services; introduce co-payments; and allow wait times to increase.^4^ But, even before the crisis, UHC was under threat in some HICs due to on-going neoliberal restructuring, the use of public-private partnerships, and the rise of commodification of health care.^4,5^ These trends have led some to argue that, rather than progressing, UHC is increasingly under attack and in actual decline in some HICs, especially in Europe.^6^

Unlike the earlier Millennium Development Goals (MDGs), the SDGs explicitly apply to all countries, and call on HICs to look inward to assess their own national situation. This presents a practical challenge as the goals (and associated monitoring mechanisms) in theory should have general applicability across all countries; in practice, however, areas like UHC do not lend themselves to common monitoring frameworks across the entire country income spectrum. The existing WHO and World Bank driven global UHC monitoring framework has little applicability in the context of HICs,^7^ which tend to uniformly score high values in the proposed index of essential health services. An appropriate UHC monitoring framework for HICs nonetheless is crucial to ensure that HICs do not backtrack on their SDG commitments at home.

As a first step towards developing a UHC monitoring framework meaningful for HICs, this short communication presents preliminary findings on the extent to which HICs are already monitoring progress in the area of health service and population coverage. It offers considerations to guide meaningful UHC monitoring in HICs and reflects on pertinent challenges and tensions to inform future research on UHC implementation in HIC settings.

## Methods

For the purpose of this study, 78 HICs were identified based on the World Bank Group classifications as of June 2017 (see List 1 in Supplementary file 1). In January 2018, we collected and analyzed key documents from 4 bodies of literature pertaining to UHC monitoring: voluntary national reviews (VNRs) of SDG implementation by HICs; national health reports by HICs; academic articles about UHC monitoring in HICs; and peer-reviewed original research articles studying health interventions in the context of UHC in HICs (see Table).

**Table:** Methods Used to Collect and Analyze Documents About UHC Monitoring From 4 Bodies of Literature

**Body of Literature**	**Method of Collecting Documents**	**Analysis Approach**
VNRs of SDG implementation	We retrieved all publicly available HIC VNRs of SDG implementation published in English or Spanish as of January 2018 (n = 15) on the United Nations Sustainable Development Knowledge Platform website (https://sustainabledevelopment.un.org/vnrs/) (see List 2 in Supplementary file 1).	Two researchers read all documents and extracted information about how UHC is discussed, whether UHC was framed as a domestic or international concern, and what indicators are used by countries in their assessment of progress towards achieving UHC.
National health reports	We gathered all publicly available national health reports by HICs published in English since 2015 available publicly on government websites (n = 24) (see List 3 in Supplementary file 1).	We performed a document analysis of all publicly available national health reports to summarize the main themes and indicators related to UHC.
Academic articles about UHC monitoring in HICs (including reviews, original research and conceptual articles and commentaries)	In January 2018 we collected English-language academic articles specifically addressing UHC monitoring in HICs, published in English since 2000 and indexed in the PubMed database. We first performed a search using Boolean operators: (“Universal health coverage”) AND monitoring AND (“high-income” OR “high income”). This search yielded 9 results with few relevant findings. We then broadened the search terms to: (“Universal health coverage”) AND (monitoring or measuring), which yielded 117 hits and contained relevant material. We screened the abstracts based on the following inclusion criteria: article must discuss monitoring of UHC in a high-income context; (or) article must make a conceptual contribution relevant to monitoring of UHC in high-income contexts; (and) article was published in English after 2000. This yielded 17 relevant articles. We included additional articles that were referenced in the reviewed articles and contained relevant information (n = 7), for a total number of 24 articles reviewed.	We conducted a narrative literature review, using the WHO UHC framework to organize our empirical findings (which distinguishes between coverage of health services, coverage of financial protection, and population coverage). We also identified general principles and best practices recommended for effective UHC monitoring applicable to HIC settings.
Peer-reviewed original research articles studying health interventions in the context of UHC in HICs	In January 2018 we conducted a systematic search of peer-reviewed research articles indexed in PubMed database to identify characteristics of research addressing health interventions in the context of UHC in HICs. Search terms were related to HICs and UHC, and included a combination of medical subject heading (mesh) terms and keywords in the article title or abstract, combined using Boolean operators “AND” and “OR.” Filters were applied to limit results to articles published since 2000, in English (see Table S1 in Supplementary file 1). The initial search yielded 617 results. Two researchers reviewed article titles (n = 617), abstracts (n = 483), and then full texts (n = 134), applying the following inclusion criteria: covers one or more HIC; addresses at least one health intervention indicator; monitors the intervention in a quantitative manner, primarily drawing from empirical data (rather than, for example, mathematical modelling); and addresses one or more aspects of coverage, use, quality or effectiveness. A total of n = 114 articles were included.	We developed and applied a rubric for the extraction of key data from the included articles. Major categories for data extraction included: study setting and population; health intervention indicators; dimensions of inequality and data sources. Key characteristics of the articles were compiled in a spreadsheet, and then summarized across studies. The goal of this review was to identify data sources for health interventions in HICs that could be used to track monitoring of UHC.

Abbreviations: UHC, universal health coverage; VNRs, voluntary national reviews; SDG, Sustainable Development Goal; HIC, high-income country; WHO, World Health Organization.

These 4 bodies of literature represent distinct sources of information on the topic, and from each body of literature we extracted relevant information. VNRs of SDG implementation and national health reports capture public and official government reports on SDG implementation and national health programs, respectively, and permit an assessment of how/if countries mention UHC in their national health reports and which indicators are used to present the health status of the population. Academic articles were sourced to explore current discussions and debates about UHC monitoring in HICs, from which we identified general principles and best practices recommended for effective UHC monitoring. Since this literature is very limited, we also analyzed peer-reviewed original research articles that provide an overview of the characteristics of research on health interventions conducted since 2000 relevant to the context of UHC. We systematically extracted and compiled key data about study settings and populations, health intervention indicator(s), dimensions of inequality, and data sources. This compilation allowed us to identify additional potential sources of data for UHC monitoring.

## Results

### UHC Progress Monitoring Is Largely Absent From VNRs

An analysis of all HIC VNRs of SDG implementation available by January 2018 (n = 15) reveals that only half of the reports mention UHC at all. Of the reports that directly mention UHC, only 3 describe indicators that are used nationally to monitor health coverage (Denmark, Germany, and France), but none specifically mention how they will measure achievement of UHC. Some countries acknowledge the domestic importance of UHC (Italy and Cyprus) but fail to propose any strategy for how to achieve it. But, the majority of HICs discuss UHC predominantly in the context of promoting achievement of UHC in LMICs, and what HICs can do to contribute to this endeavor through development assistance. Japan is a prominent example of this international (as opposed to domestic) focus on UHC, as it notes that its own experiences with a well-functioning universal health insurance system mean that it can play a leadership role in the promotion of UHC in low-income countries.

In addition to the SDG reports, we sought out HIC government-issued health reports to identify the role of UHC in national health repots and determine whether the importance of UHC is reflected in them. While not all HIC issue such (often annual or bi-annual) health reports to track progress, we found 24 publicly available reports by HICs published since 2015 and analyzed them for discussion of UHC implementation. Only 3 countries (Brunei Darussalam, Oman, and Qatar) explicitly mention UHC in their health reports and do so only in terms of claiming to have fully achieved UHC. However, even though most countries did not explicitly mention UHC, we found that some of the 16 UHC tracer indicators proposed by WHO^8^ are indeed used by HICs when discussing the current health status of their population. Countries also use a wide range of additional health indicators, which might represent more appropriate indicators in monitoring progress of realization of UHC in high-income settings, to which we return in the discussion section (see Table S2 in Supplementary file 1 for a full list of health indicators compiled from 24 national health reports).

### HICs Require Different Monitoring Approaches than LMICs

Academic discussions on UHC monitoring focus predominantly on LMICs, amidst acknowledgment that the WHO proposed monitoring framework is largely irrelevant for HICs.^8^ The health coverage service index, developed and used by WHO, combines 16 tracer indicators, including 4 from within each of the categories of reproductive, maternal, newborn, and child health; infectious disease; non-communicable diseases (NCDs); and service capacity and access. Indicator data for 183 countries are collected from UN agency estimates or databases, supplemented with submissions from national focal points during WHO country consultations. The index is computed using geometric means, and a subset of tracer indicators is used to summarise inequalities.^8^ While this index represents a welcome development, especially in LMIC contexts, the index does not report on scores higher than 80, with most HICs situated in the above 80 category. But as one recent critical commentary notes: “equating the performance of Japan — a country widely lauded for its UHC accomplishments — with that of the United States, which is known for its unexceptional record in health service provision among HICs, raises other questions about how all countries, across the development spectrum, can have meaningful measures of UHC in the SDG era.”^7^ It also means that HICs are not being challenged to do better in UHC even when there may be glaring inequities in access or coverage for some populations, requiring correction and improvement. Moreover, limiting included tracer indicators in UHC monitoring to 16 indicators across 4 health domains (reproductive, maternal and neonatal, and child health; NCDs; infectious diseases; and service capacity and access) is justified by WHO in reference to data availability (based on comparable Demographic and Health Surveys) and methodological simplicity. But the decision to limit monitoring for HICs to currently available data in LMICs undermines its utility for HICs.^7^ The demands and requirements of health systems in HICs vary dramatically from LMIC settings and may require modifications that better capture and reflect levels, trends and equity of service coverage of UHC in HICs.

We found only 3 articles that directly discuss monitoring of UHC in HICs (as part of the PLOS Monitoring UHC case study series), with all 3 countries only recently moving from LMIC to HIC status.^9-11^ In the case of Chile, 2 priority areas for UHC monitoring of health service coverage are identified: the ‘unfinished business’ of the MDG health goals and targets; and reducing the burden of NCDs, on which the MDGs were stunningly silent. NCDs are the leading cause of disease in Chile, with a clear inequitable dimension in access to treatment for NCDs. Equity disaggregation shows lower coverage for males, low-income quintiles, less-educated people, and residents in rural areas. This suggests that addressing inequitable access to health interventions will be a central aspect of achieving UHC in Chile.^10^ Estonia’s UHC monitoring efforts, in turn, focus on utilizing data produced through its annual Health Systems Performance Assessment (HSPA) to measure service coverage, and to integrate quality of care as an important monitoring element.^9^ Finally, although our third case, Singapore, has no specific UHC monitoring framework in place, indicators on accessibility, quality, and affordability of health care are regularly tracked through its key performance indicators reported by the Ministry of Health.^11^ Singapore’s key performance indicators include many of the tracer indicators for both MDG related diseases and NCDs as recommended by the WHO and World Bank, with the stratified nature of access to health service coverage, an aging population, and related increases in NCDs considered the main challenges to achieving or sustaining UHC in the near future.

Apart from these 3 examples, however, none of the global UHC monitoring frameworks in the academic literature specifically addressed HIC contexts. A number of general principles did emerge in the literature that could be considered ‘best practices’ to guide the selection of indicators for monitoring of UHC in HICs. One important principle is that health interventions covered through UHC should consist of essential and good-quality services, based on “a data-rich metric to measure the extent to which an intended health benefit is provided by a specific intervention.”^12^ Such an assessment is of particular importance in HIC contexts due to mounting evidence on the magnitude of medically unnecessary (and potentially dangerous) health interventions, with the WHO specifically identifying overuse of health interventions as a potential barrier to achieving UHC.^13^

At the heart of the UHC is a commitment to improving health equity, yet there is a risk that the most vulnerable population groups in all country contexts may be left behind in UHC implementation.^14^ There is agreement in the academic literature that UHC monitoring should include multiple and complementary dimensions of inequality (such as economic status and urban/rural residence, in addition to gender). Both absolute and relative measures of inequality as well as disaggregated data should be reported, and national averages presented alongside monitoring.^15^ A key challenge in relation to equity in coverage in HICs is that inequitable health outcomes may arise even when access to primary health care is reasonably equitable, predominantly through disparities in quality of care and inequitable access to specialized clinical services^16^ due to pro-rich bias in use of specialist hospital services.^17^ Such findings led one review to conclude that in order to maximize equitable access to health interventions in HICs, UHC programs should predominantly focus on increasing coverage and decreasing economic barriers to access among the most disadvantaged segments of the population, and that monitoring should capture the impacts of affirmative actions targeted strategically at the most disadvantaged populations.^16^

Finally there are questions about the extent to which UHC monitoring systems need to integrate a broader set of social determinants of health (SDH), including upstream socio-economic environmental, and governance aspects determining population health and health equity.^18^ This is important since social gradients in health remain pervasive even in HICs with publicly financed universal health systems. Inequitable distribution of SDH can become a barrier to UHC implementation, for example, when non-medical costs related to transportation deter disadvantaged populations from accessing otherwise affordable or free health services. A SDH monitoring framework could complement the one for UHC proposed by the WHO and World Bank, which is restricted to health coverage, financial protection, and equity in access and coverage. Although not being monitored systematically at the moment, such SDH-related barriers have received mention in the first global monitoring report for UHC.^19^ Moreover, such a framework could be drawn from indicators suggested or agreed upon for monitoring of other SDG targets, many of which pertain to several SDH.^20^

### Recent Research Suggests Possible Directions for Monitoring

Given the paucity of information in VNRs and academic literature about UHC monitoring, we also analyzed the characteristics of peer-reviewed original research articles on health interventions in HICs to get an indication of aspects of research that are current and topical (namely, health intervention, dimensions of inequality and data sources). By virtue of their inclusion in this study, all articles (n = 114) framed, discussed, and/or designed the research in the context of UHC. This UHC framing of health interventions research in HICs has been on a steady rise since 2000. The literature retrieved for this study reveals a 10-fold increase of articles published in 2014-2018 (n = 61 articles) as compared to articles published in 2000-2004 (n = 6), suggesting that UHC may be gaining attention among researchers. The highest proportion of the included articles pertain to health interventions in Korea (n = 13), Spain (n = 10), the United States (n = 8), Ireland (n = 7) and Switzerland (n = 7).

The majority of health interventions under study relate to curative or palliative aspects of care (n = 99), while a smaller, but substantial, number focus on promotive or preventive interventions (n = 29). Some commonly studied aspects of curative and palliative interventions include physician consultations, secondary care services, treatment regimes, and hospital admissions. Promotive and preventive interventions include disease screening, disease risk factor reduction, and vaccination. While most articles report health indicators of service use (n = 95), several also address quality issues (n = 49; examples include health worker density and the receipt of appropriate interventions for a given condition). A number of articles (n = 21) address intervention effectiveness, showing the extent to which the intervention produced the desired result (for example, recovery time after surgery, satisfaction assessments, and avoidable hospital admissions).

The selection and application of dimensions of inequality – the criteria by which data are disaggregated for equity-sensitive monitoring – is an important aspect of how population access to UHC is conceptualized (ie, which population “gaps” in access are being measured^21^). In our sample, most of the research articles disaggregate health indicator data by one or more dimension of inequality. These include (in descending order of frequency mentioned): age, sex/gender, economic status, education status, insurance status, marital status, nationality/immigrant status/place of birth, employment, ethnicity/race, subnational region, and urban/rural place of residence. While the categorization of certain dimensions is relatively straightforward (eg, age and gender), others, such as economic status, necessitate context-specific approaches in HICs, due to the applicability of the metrics to measure the construct (eg, in countries with a large informal sector, economic status may be more appropriately captured through household assets than reported income). In the 52 research articles that disaggregate health data by economic status, there is variability in how such status was measured: while most studies measured economic status as income (at the individual, household or small geographical area level), a fewer number of studies applied deprivation indices to small geographical areas or less commonly, relied on household assets or consumption.

Nearly half of studies (n = 54) drew health-related data from institution-based records, including insurance, medical, administrative, and municipal records. Institution-based sources were also commonly used for dimensions of inequality data. The second-most common data source was household surveys, followed by primary data sources generated for research purposes (eg, questionnaires or interviews). A smaller number of research articles reported data from multinational databases (namely, databases of United Nations organizations), census data, and disease surveillance systems.

## Discussion and Conclusion

Effectively monitoring UHC in high-income settings remains a central challenge in the SDG process, with no simple solutions on offer. But given the centrality of UHC on the global health stage, and its ascent in global policy dialogue, including as a pillar of the WHO Global Programme of Work for 2019-2023, there is a need for better monitoring tools for use in HICs than those that are currently available.^7^ National health inequality monitoring, an application of population health monitoring that tracks the performance of disadvantaged population subgroups over time with respect to a specified health indicator, is essential across all country contexts to measure the progressive realization of UHC and guide equity-oriented policy-making.^22,23^ The broader convergence agenda of the SDGs, including promoting UHC in all countries, may one day create opportunities for developing cross-country best practices, especially as LMICs make variable progress towards UHC and some HICs risk backsliding. However, currently the specifics of monitoring – the selection of relevant health indicators, dimensions of inequality, data sources, and analysis and reporting approaches – are often different in HICs than LMICs.

Generally, as the academic literature finds, the data sources available for UHC monitoring in HICs are more detailed, diverse and complex than those available for LMICs, and go beyond those integrated into the UHC monitoring framework proposed by WHO. Institution-based records, in particular, are a prominent source of data in recent research articles. With the roll-out and refinement of eHealth technologies across HICs, health records become a rich source of institution-based data for monitoring that, due to availability in digital and increasingly standardized formats, provide pertinent health and sociodemographic data. Equally, data derived from health insurance claims and other administrative sources are increasingly available in digital formats that could be harnessed for UHC monitoring purposes. While data sources in HICs offer a multitude of options for monitoring, the comparability of the sources between – and sometimes within – may be limited. With the establishment of clear national frameworks for UHC monitoring, targeted action could then be taken to make current data sources more fit-for-purpose and comparable.

An important consideration of UHC monitoring in HICs relates to the integration of such monitoring within existing national health reports and the wider reporting infrastructure, especially regular and on-going health systems performance reviews conducted regularly by HICs. One possible solution would be to develop a comprehensive set of core UHC indicators specifically for HICs which can be adapted to each country situation, and reviewed on a regular basis as part of health system performance assessments.^12^ Ideally, countries would embed any monitoring activities within the WHO health performance review process already in place to reduce administrative burden and enhance dissemination of findings to hold countries accountable. As noted above, this is the case in Estonia, which did not develop a UHC-specific monitoring framework but rather assessed progress towards achieving UHC as part of its HSPA. HSPAs represent a common conceptual framework developed by WHO for health systems performance assessment, to encourage the development of tools to measure its components, and to collaborate with countries in applying these tools to measure and then to improve health systems performance. The existing reporting infrastructure in most HICs could facilitate an integration of UHC monitoring within their existing health performance reviews.

It is clear that the measurement of service coverage needs to go beyond the currently proposed 14 UHC indicators but it is less clear what specific health interventions should be privileged and tracked to implement progressive realization of UHC in HICs. The proper fit between a country’s unique epidemiological and demographic make-up and indicators selected to measure progress towards achieving UHC is central to establishing meaningful domestic monitoring frameworks. An evidence based deliberative approach could start from the epidemiological profile of a country to identify key health challenges, as was the case in Chile discussed above. It next would need to identify what health interventions to track based on their importance to the health profile of the country.^24^

Further, our findings suggest that in order to maximize equitable access to health interventions in HICs, UHC programs should predominantly focus on increasing coverage and decreasing economic barriers to access amongst the most disadvantaged segments of the population. This begs the question of how to identify and define disadvantaged subgroups, which currently tend to be based on age, sex/gender, and economic status; these determinations, however, are dynamic between contexts and over time, reflecting relevant (or possible) sources of discrimination within a population. Monitoring should capture the extent to which affirmative actions targeted strategically at the most disadvantaged populations are successful. For example, HICs could monitor expansion of essential health services to hard-to-reach senior populations given the growing challenge of equitable access to health services by seniors in aging HIC populations.^14^ In addition, quality based indicators (eg, effective coverage) are increasingly integrated into health system monitoring^25^; they are particularly pertinent for measuring achievement of UHC in the realm of service coverage in HICs, given that many countries have largely achieved near universal contact coverage for basic service interventions, and that inequitable health outcomes often relate to quality of service, with higher income individuals receiving better quality care for the same condition.

Progressive realization of UHC implies that it cannot ever be fully achieved as such, and that there will always be room to expand coverage of health services. This flags the need for informed debate about the normative underpinnings of equitable coverage. Monitoring approaches, however much improved or expanded to account for different economic contexts, nonetheless need to be flexible and specific to the priorities of the populations that they serve. The biggest challenge is the general tension between developing a universal framework for monitoring that encompasses all countries (and permits comparability and benchmarking), versus country-specific approaches that allow for contextual considerations and have greater applicability in-country. Ultimately, we argue the need for both while acknowledging that developing and applying a universal UHC framework relevant to all HICs will be difficult to achieve. This is less likely to be an issue of insufficient knowledge or data, but more consequent to a lack of consensus amongst such nations about how to measure UHC which, in turn, is linked to a lack of political interest by many HICs to take the SDGs seriously, at least as applied to their own domestic settings. The choice of a WHO reporting cut-off at the 80 index point is indicative of the fact that HICs did not want to be scored by WHO on UHC progress. As long as the unwillingness of HICs to apply the SDGs domestically prevails, little progress can be expected in developing a UHC monitoring framework ‘fit-for-purpose’ in high-income contexts. This is why going forward, research on UHC monitoring should also engage with the political barriers, and the wider political economy context within which discussions of UHC monitoring take place.^26^

## Acknowledgements

The research was funded through a research contract (#201833637) with the WHO but the findings exclusively represent the views of the authors and do not in any way reflect the views of WHO. We would like to acknowledge the research assistance of Layal Alessandra Mounzer and Nura Mesoud who contributed to the empirical data analysis.

## Ethical issues

Not applicable.

## Competing interests

Authors declare that they have no competing interests.

## Authors’ contributions

NB and AR were the grant holders for this research. They conducted the research and drafted the paper. RL provided substantial suggestions and comments throughout the process of drafting the final version of the manuscript. All authors read and approved the final manuscript.

## Authors’ affiliations

^1^Faculty of Health Sciences, University of Ottawa, Ottawa, ON, Canada. ^2^Faculty of Medicine, School of Epidemiology and Public Health, University of Ottawa, Ottawa, ON, Canada. ^3^Canada Research Chair, Globalization and Health Equity, Faculty of Medicine, School of Epidemiology and Public Health, University of Ottawa, Ottawa, ON, Canada.

## Supplementary files


Supplementary file 1 contains:
**List 1.** High-Income Countries According to World Bank Classification in June 2017.
**List 2.** High-Income Country Voluntary National Reviews Available from the United Nations Sustainable Development Knowledge Platform in January 2018.
**List 3.** List of High-Income Country National Health Reports, gathered in January 2018 from Publicly Available Government Websites.
**Table S1.** Search Strategy for Retrieval of Peer-Reviewed Original Research Articles of Health Interventions in the Context of Universal Health Coverage in High Income Countries.
**Table S2.** Health Indicators Compiled from 24 National Health Reports.
