# Supplementary material for: Monitoring Frameworks for Universal Health Coverage: What About High-Income Countries?
Source: Int J Health Policy Manag. 2019 Apr 22;8(7):387–93. doi: 10.15171/ijhpm.2019.21 (PMC6706973; doi:10.15171/ijhpm.2019.21)
Supplement: Supplementary file 1 — contains: List 1. High-Income Countries According to World Bank Classification in June 2017. List 2. High-Income Country Voluntary National Reviews Available from the United Nations Sustainable Development Knowledge Platform in January 2018. List 3. List of High-Income Country National Health Reports, gathered in January 2018 from Publicly Available Government Websites. Table S1. Search Strategy for Retrieval of Peer-Reviewed Original Research Articles of Health Interventions in the Context of Universal Health Coverage in High Income Countries. Table S2. Health Indicators Compiled from 24 National Health Reports. [file ijhpm-8-387-s001.pdf]

Supplementary file 1

List 1. High Income Countries According to World Bank Classification in June 2017

Andorra, Antigua and Barbuda, Aruba, Australia, Austria, the Bahamas, Bahrain, Barbados, Belgium, Bermuda, British Virgin Islands, Brunei Darussalam, Canada, Cayman Islands, Channel Islands, Chile, Croatia, Curacao, Cyprus, Czech Republic, Denmark, Estonia, Faroe Islands, Finland, France, French Polynesia, , Germany, Gibraltar, Greece, Greenland, Guam, Hong Kong (SAR, China), Hungary, Iceland, Ireland, Isle of Man, Israel, Italy, Japan, Republic of Korea, Kuwait, Latvia, Liechtenstein, Lithuania, Luxembourg, Macao (SAR, China), Malta, Monaco, Nauru, Netherlands, New Caledonia, New Zealand, Northern Mariana Islands, Norway, Oman, Poland, Portugal, Puerto Rico, Qatar, San Marino, Saudi Arabia, Seychelles, Singapore, Sint Maarten (Dutch part), Slovak Republic, Slovenia, Spain, St. Kitts and Nevis, St. Martin (French part), Sweden, Switzerland, Taiwan (China), Trinidad and Tobago, Turks and Caicos Islands, United Arab Emirates, United Kingdom, United States of America, Uruguay, Virgin Islands (U.S.).

List 2. High Income Country Voluntary National Reviews Available from the United Nations Sustainable Development Knowledge Platform in January 2018

Belgium, Chile, Czech Republic, Denmark, Estonia, Finland, France, Germany, Japan, Luxembourg, Portugal, Monaco, Slovenia, Sweden, Uruguay.

List 3. List of High Income Country National Health Reports, gathered in January 2018 from Publicly Available Government Websites

Australia, Bahrain, Bermuda, Brunei Darussalam, Canada, Cayman Islands, Channel Islands, Croatia, Denmark, Estonia, Germany, Gibraltar, Hong Kong (SAR, China), Ireland, Israel, Nauru, Oman, Qatar, Saudi Arabia, Singapore, Spain, Taiwan, United Kingdom, United States of America.

Table S1. Search Strategy for Retrieval of Peer-Reviewed Original Research Articles of Health Interventions in the Context of Universal Health Coverage in High Income Countries

Description of search category	PubMed search terms
High income countries terms	ANDORRA[MESH] OR “ANTIGUA AND BARBUDA”[MESH] OR ARUBA[MESH] OR AUSTRALIA[MESH] OR AUSTRIA[MESH] OR BAHAMAS[MESH] OR BAHRAIN[MESH] OR BARBADOS[MESH] OR BELGIUM[MESH] OR BERMUDA[MESH] OR “BRITISH VIRGIN ISLANDS”[MESH] OR “BRUNEI DARUSSALAM”[MESH] OR CANADA[MESH] OR “CAYMAN ISLANDS”[MESH] OR “CHANNEL ISLANDS”[MESH] OR CHILE[MESH] OR CROATIA[MESH] OR CURACAO[MESH] OR CYPRUS[MESH] OR “CZECH REPUBLIC”[MESH] OR DENMARK[MESH] OR ESTONIA[MESH] OR FAROE ISLANDS[MESH] OR FINLAND[MESH] OR FRANCE[MESH] OR “FRENCH POLYNESIA”[MESH] OR GERMANY[MESH] OR GIBRALTAR[MESH] OR GREECE[MESH] OR GREENLAND[MESH] OR GUAM[MESH] OR “HONG KONG”[MESH] OR HUNGARY[MESH] OR ICELAND[MESH] OR IRELAND[MESH] OR “ISLE OF MAN”[MESH] OR ISRAEL[MESH] OR ITALY[MESH] OR JAPAN[MESH] OR KOREA[MESH] OR KUWAIT[MESH] OR LATVIA[MESH] OR LIECHTENSTEIN[MESH] OR LITHUANIA[MESH] OR LUXEMBOURG[MESH] OR “MACAO”[MESH] OR MALTA[MESH] OR MONACO[MESH] OR NAURU[MESH] OR NETHERLANDS[MESH] OR “NEW CALEDONIA”[MESH] OR “NEW ZEALAND”[MESH] OR “NORTHERN MARIANA ISLANDS”[MESH] OR NORWAY[MESH] OR OMAN[MESH] OR POLAND[MESH] OR PORTUGAL[MESH] OR “PUERTO RICO”[MESH] OR QATAR[MESH] OR “SAN MARINO”[MESH] OR “SAUDI ARABIA”[MESH] OR SEYCHELLES[MESH] OR SINGAPORE[MESH] OR “SINT MAARTEN”[MESH] OR “SLOVAK REPUBLIC”[MESH] OR SLOVENIA[MESH] OR SPAIN[MESH] OR “ST. KITTS AND NEVIS”[MESH] OR “ST. MARTIN”MESH] OR SWEDEN[MESH] OR SWITZERLAND[MESH] OR “TRINIDAD AND TOBAGO”[MESH] OR “TURKS AND CAICOS ISLANDS”[MESH] OR “UNITED ARAB EMIRATES”[MESH] OR “UNITED KINGDOM”[MESH] OR “UNITED STATES”[MESH] OR URUGUAY[MESH] OR “VIRGIN ISLANDS”[MESH] OR "DEVELOPED COUNTRIES"[MESH] OR ANDORRA[TIAB] OR “ANTIGUA AND BARBUDA”[TIAB] OR ARUBA[TIAB] OR “AUSTRALIA”[TIAB] OR AUSTRIA[TIAB] OR BAHAMAS[TIAB] OR BAHRAIN[TIAB] OR BARBADOS[TIAB] OR BELGIUM[TIAB] OR BERMUDA[TIAB] OR “BRITISH VIRGIN ISLANDS”[TIAB] OR BRUNEI[TIAB] OR CANADA[TIAB] OR “CAYMAN ISLANDS”[TIAB] OR “CHANNEL ISLANDS”[TIAB] OR CHILE[TIAB] OR CROATIA[TIAB] OR CURACAO[TIAB] OR CYPRUS[TIAB] OR “CZECH REPUBLIC” TIAB] OR DENMARK[TIAB] OR ESTONIA[TIAB] OR “FAROE

	ISLANDS"[TIAB] OR FINLAND[TIAB] OR FRANCE[TIAB] OR "FRENCH POLYNESIA"[TIAB] OR GERMANY[TIAB] OR GIBRALTAR[TIAB] OR GREECE[TIAB] OR GREENLAND[TIAB] OR GUAM[TIAB] OR HOLLAND[TIAB] OR "HONG KONG"[TIAB] OR HUNGARY[TIAB] OR ICELAND[TIAB] OR IRELAND[TIAB] OR "ISLE OF MAN"[TIAB] OR ISRAEL[TIAB] OR ITALY[TIAB] OR JAPAN[TIAB] OR KOREA[TIAB] OR KUWAIT[TIAB] OR LATVIA[TIAB] OR LIECHTENSTEIN[TIAB] OR LITHUANIA[TIAB] OR LUXEMBOURG[TIAB] OR MACAO[TIAB] OR MALTA[TIAB] OR MONACO[TIAB] OR NAURU[TIAB] OR NETHERLANDS[TIAB] OR "NEW CALEDONIA"[TIAB] OR "NEW ZEALAND"[TIAB] OR "NORTHERN MARIANA ISLANDS"[TIAB] OR NORWAY[TIAB] OR OMAN[TIAB] OR POLAND[TIAB] OR PORTUGAL[TIAB] OR "PUERTO RICO"[TIAB] OR QATAR[TIAB] OR "SAN MARINO"[TIAB] OR "SAUDI ARABIA"[TIAB] OR SEYCHELLES[TIAB] OR SINGAPORE[TIAB] OR "SINT MAARTEN"[TIAB] OR "SLOVAK REPUBLIC"[TIAB] OR SLOVENIA[TIAB] OR SPAIN[TIAB] OR "ST. KITTS AND NEVIS"[TIAB] OR "ST. MARTIN"[TIAB] OR SWEDEN[TIAB] OR SWITZERLAND[TIAB] OR "TRINIDAD AND TOBAGO"[TIAB] OR "TURKS AND CAICOS ISLANDS"[TIAB] OR "UNITED ARAB EMIRATES"[TIAB] OR "UNITED KINGDOM"[TIAB] OR "UNITED STATES"[TIAB] OR URUGUAY[TIAB] OR "VIRGIN ISLANDS"[TIAB] OR "DEVELOPED COUNTRIES"[TIAB] OR "DEVELOPED COUNTRY"[TIAB] OR "HIGH-INCOME COUNTRIES"[TIAB] OR "HIGH INCOME COUNTRIES "[TIAB] OR "HIGH-INCOME COUNTRY"[TIAB] OR "HIGH INCOME COUNTRY"[TIAB] OR "FIRST WORLD COUNTRIES"[TIAB]
	AND
Universal health coverage terms	"UNIVERSAL COVERAGE"[MESH] OR "UNIVERSAL COVERAGE"[TIAB] OR "UNIVERSAL HEALTH COVERAGE"[TIAB] OR "UNIVERSAL CARE"[TIAB] OR "UNIVERSAL HEALTH CARE COVERAGE"[TIAB] OR "UNIVERSAL HEALTH CARE"[TIAB] OR "UNIVERSAL HEALTH SYSTEM"[TIAB]
Note: search filters were applied, limiting the search to English language articles published between January 1, 2000 and January 25, 2018	

Table S2. Health Indicators Compiled from 24 National Health Reports

Tracer Area	Tracer Indicator	Number of Countries	Countries
**REPRODUCTIVE, MATERNAL, NEWBORN AND CHILD HEALTH**	Percentage of women who were overweight when they became pregnant	3	Germany, Channel Islands
	Receipt of full series of HPV for both male and female adolescents (ages 13-17)	2	Australia, USA
	Percentage of children and adolescents (5-19) with untreated dental caries	2	Germany, USA
	The percentage of infants aged 6 months that are partly or fully on breast feeding	6	Croatia, Estonia Germany, Qatar, Taiwan, USA
	The percentage of adolescents (2-19 years old) that are obese	5	Australia, Bermuda, Channel Islands, Taiwan, USA
	The percentage of adolescents under 18 with a food or skin allergy	5	Australia, Croatia, Germany, Ireland, USA
	Percentage of children and adolescents (5-17) diagnosed with ADHD	2	Australia, USA
**INFECTIOUS DISEASES**	Influenza vaccination rates for the elderly aged 65 year+	8	Australia, Bermuda, Estonia, Germany, Israel, Singapore, Spain, USA
	Vaccination rates for pneumococcal disease	1	Australia

	Mortality rates from pneumonia in older people	2	Australia, Bermuda
	Number of MRSA cases after more than 48 hours of admission	2	Australia, Singapore
**NON-COMMUNICABLE DISEASES**	In hospital mortality following acute myocardial infarction (AMI).	1	Bermuda
	Prevalence of adults with high cholesterol.	8	Australia, Bermuda, Brunei Darussalam, Gibraltar, Hong Kong, Israel, Spain, USA
	Percentage of smokers trying to quit	1	Ireland
	Prevalence of adolescents that report smoking e-cigarettes	3	Australia, Canada, USA
	Drug poisoning death rate involving heroin or opioid analgesics.	1	USA
	Number of people treated for drug abuse	1	Croatia
	Colorectal cancer screening.	7	Australia, Brunei Darussalam, Croatia, Denmark, Gibraltar, Israel, USA
	Breast cancer Screening	10	Australia, Bermuda, Brunei Darussalam, Croatia, Denmark,

			Germany, Gibraltar, Israel, Spain, USA
	The proportion of adults obese or overweight	9	Australia, Bermuda, Brunei Darussalam, Channel Islands, Croatia, Estonia, Israel, Taiwan, USA
	Percentage of people with anxiety disorders, depression, sleep disorders and alcoholism	4	Australia, Channel Islands, Croatia, Germany
	Percentage of People with dementia	2	Australia, Germany
	Percentage of people who have a partner or a close family member that has/had dementia	1	Ireland
	Number of new cases of treated end-stage kidney disease (ESKD) or end-stage renal failure (ESRF)	4	Australia, Bermuda, Israel, Saudi Arabia
	Percentage of people who sometimes or always need help with core activities of daily living (mobility, self-care or communication)	1	Australia
	Percentage of adults with pain in the lumbar spine, osteoarthritis or cervical back pain	4	Australia, Germany, Spain, USA

**SERVICE CAPACITY AND ACCESS**	The average experienced waiting time for planned hospital surgery	2	Australia, Denmark
	Average wait time for patients who are to have an initial consultation at the specialised care level.	2	Australia, Spain
	Average length of stay (ALOS)	3	Australia, Bermuda, USA
	The percentage of people who self-assess their health as excellent or very good.	4	Australia, Bermuda, Estonia, Spain
**SOCIAL DETERMINANTS OF HEALTH**	The percentage of people that say they have a somewhat or a very strong sense of community belonging.	1	Canada
	Health literacy level above the minimum level regarded as necessary for understanding and using information relating to health issues.	2	Australia, Canada
	Sea quality, air quality and soil quality.	2	Australia, Croatia
